# Increased prolyl endopeptidase induces alterations in the expression of neural cell adhesion molecule and its polysialylation in SH-SY5Y neuroblastoma cells in vitro

**DOI:** 10.1186/2193-1801-4-S1-P49

**Published:** 2015-06-12

**Authors:** Keiti Uibokand, Külli Jaako, Aveli Noortoots, Anti Kalda, Alexander Zharkovsky

**Affiliations:** Institute of Biomedicine and Translational Medicine, Department of Pharmacology, University of Tartu, Estonia

**Keywords:** Neural cell adhesion molecule, prolyl endopeptidase, cellular plasticity

Development of the nervous system and its structural remodelling in the adult relies on molecules mediating the structural plasticity of neurons, e specially those involved in cell adhesion, cytoskeletal dynamics or synapse formation. Neural cell adhesion molecule (NCAM) is a membrane-associated glycoprotein that can be modified by glycosylation with polysialic acid (PSA), attenuating NCAM-mediated cell interactions, thereby promoting structural plasticity [1]. It has been demonstrated that in conditions of neuroinflammation, NCAM can be cleaved extracellularly by metalloproteinases and other proteolytical enzymes. Prolyl endopeptidase (PREP) is a cytosolic serine protease, and alterations in PREP expression and activity have been associated with neuronal death and neuroinflammation [2]. Since the precise mechanisms and possible partners of PREP in neuroinflammation remain unclear, the aim of this study was to determine whether increased secretion and activation of PREP could impair NCAM expression and its polysialylation. SH-SY5H cell line overexpressing (o/e) PREP was used as an in vitro model for increased PREP expression and extracellular release. When measuring expression levels of NCAM and PSA-NCAM we found remarkable loss in PSA-NCAM and disrupted expression patterns of NCAM compared to wild-type cells. As matrix metalloproteinase 9 (MMP-9) has been indicated in processes regulating shedding of PSA-NCAM, MMP-9 expression level was measured. An increase in the level of the active form of MMP-9 was found, which was counteracted by a specific inhibitor of PREP, KYP-2047. As demonstrated, PREP might have an important role in processes involved in NCAM degradation and polysialylation, thereby inducing progression of pathologies associated with altered neuroplasticity.
